# High-Throughput Sequencing Reveals a Potentially Novel *Sulfurovum* Species Dominating the Microbial Communities of the Seawater–Sediment Interface of a Deep-Sea Cold Seep in South China Sea

**DOI:** 10.3390/microorganisms8050687

**Published:** 2020-05-08

**Authors:** Qing-Lei Sun, Jian Zhang, Min-Xiao Wang, Lei Cao, Zeng-Feng Du, Yuan-Yuan Sun, Shi-Qi Liu, Chao-Lun Li, Li Sun

**Affiliations:** 1 CAS Key Laboratory of Experimental Marine Biology, CAS Center for Ocean Mega-Science, Institute of Oceanology, Chinese Academy of Sciences, Qingdao 266071, China; sunqinglei@qdio.ac.cn (Q.-L.S.); zhangjian@qdio.ac.cn (J.Z.); sunyuanyuan@qdio.ac.cn (Y.-Y.S.); 2 Laboratory for Marine Biology and Biotechnology, Qingdao National Laboratory for Marine Science and Technology, Qingdao 266071, China; 3 Deep Sea Research Center, Institute of Oceanology, Chinese Academy of Sciences, Qingdao 266071, China; wangminxiao@qdio.ac.cn (M.-X.W.); caolei@qdio.ac.cn (L.C.); duzengfeng@qdio.ac.cn (Z.-F.D.); 4 CAS Key Laboratory of Marine Ecology and Environmental Sciences, Institute of Oceanology, Chinese Academy of Sciences, Qingdao 266071, China; 5 Key Lab of Marine Geology and Environment, Institute of Oceanology, Chinese Academy of Sciences, Qingdao 266071, China; 6 Faculty of Science, University of Amsterdam, 1098XH Amsterdam, The Netherlands; s.liu2@uva.nl; 7 University of Chinese Academy of Sciences, Beijing 100049, China

**Keywords:** cold seep, seawater–sediment interface, sulfur-oxidizing bacteria, Campylobacterota, *Sulfurovum*

## Abstract

In the Formosa cold seep of the South China Sea (SCS), large amounts of methane and sulfide hydrogen are released from the subseafloor. In this study, we systematically investigated the microbial communities in the seawater–sediment interface of Formosa cold seep using high-throughput sequencing techniques including amplicon sequencing based on next-generation sequencing and Pacbio amplicon sequencing platforms, and metagenomics. We found that *Sulfurovum* dominated the microbial communities in the sediment–seawater interface, including the seawater close to the seepage, the surface sediments, and the gills of the dominant animal inhabitant (*Shinkaia crosnieri*). A nearly complete 16S rRNA gene sequence of the dominant operational taxonomic units (OTUs) was obtained from the Pacbio sequencing platforms and classified as OTU-L1, which belonged to *Sulfurovum*. This OTU was potentially novel as it shared relatively low similarity percentages (<97%) of the gene sequence with its close phylogenetic species. Further, a draft genome of *Sulfurovum* was assembled using the binning technique based on metagenomic data. Genome analysis suggested that *Sulfurovum* sp. in this region may fix carbon by the reductive tricarboxylic acid (rTCA) pathway, obtain energy by oxidizing reduced sulfur through sulfur oxidizing (Sox) pathway, and utilize nitrate as electron acceptors. These results demonstrated that *Sulfurovum* probably plays an important role in the carbon, sulfur, and nitrogen cycles of the Formosa cold seep of the SCS. This study improves our understanding of the diversity, distribution, and function of sulfur-oxidizing bacteria in deep-sea cold seep.

## 1. Introduction

Large amounts of methane in solid, dissolved, and gas forms have been deposited in subsurface seabeds of continental margins [1]. Cold seeps refer to the sites where methane rises up to the seafloor. Cold seeps were first discovered in the Florida Escarpment in the Gulf of Mexico in the 1980s [2,3], and since then a large number of cold seeps have been found in the seafloors around the world. Cold seep is viewed as a significant pathway for Earth’s material recycling, and the release of methane (CH_4_) and other chemical compounds is essential to sustain the local seafloor ecosystem [4]. Generally, dense biological communities exist in cold seeps, like “oasis” in the seafloor. Microbes play a vital role in cold seeps and are involved in important reactions, such as anaerobic oxidation of methane (AOM) [5]: CH_4_ (aq) + SO_4_^2−^ → HS^−^ + HCO_3_^−^ + H_2_O. Under the subseafloor of cold seep, anaerobic methane-oxidizing archaea (ANME) can perform AOM alone or in partnership with sulfate reducing bacteria. The diversity and distribution of ANME have been well studied in many cold seeps [6,7,8,9,10]. To date, three types of ANME, namely, ANME-1, -2, and -3, have been described [8,11]. However, neither ANME nor their partners, sulfate reducing bacteria, have been isolated from cold seep. On the seafloor of cold seep, methane-oxidizing bacteria (MOB) are abundant [12,13,14]; however, only one bacterial strain, *Methyloprofundus sediment*, was successfully isolated from cold seep [15].

Methane oxidization is known to be accompanied with H_2_S release. Previous studies have indicated that sulfur-oxidizing bacteria (SOB), including Campylobacterota (previously known as Epsilonproteobacteria) [16,17,18] and Gammaproteobacteria, inhabited deep-sea cold seep sediments and animals [19,20,21]. However, the diversity and distribution of SOB are unclear in most cold seeps around the world, owing to the scarcity of studies on these bacteria. As a result, the functions and roles of SOB in cold seeps are probably underestimated.

Many seep sites have been discovered in the South China Sea (SCS) continental slope, including Formosa Ridge and Haima, where active seepage and dense animal communities have been observed [22]. In 2017 and 2018, two scientific cruises in Formosa cold seep were carried out, during which, high concentrations of methane and sulfide hydrogen in the seepage seawater and sediments were detected, and sediment, animal, and seawater samples were obtained. In the present study, we utilized these samples and examined the microbial community structure of the sediment–seawater interface using next-generation sequencing (NGS) and the third-generation sequencing techniques. We determined the main functional community and obtained the draft genome of high-abundance bacterium to assess their roles in the cold seep using binning technique based on metagenomics.

## 2. Materials and Methods

### 2.1. Sites and Sampling

**Figure 1:**
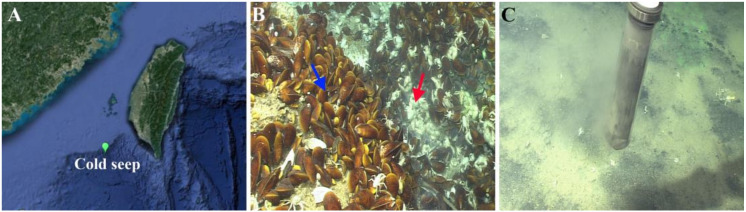
The location of and the sampling area in the cold seep. (**A**) The location of the cold seep is indicated. (**B**) The representative animal communities in the cold seep. *Bathymodiolus platifrons* and *Shinkaia crosnieri* are indicated with the blue and red arrows, respectively. (**C**) Sediment samples were collected using a pushcore.

Samples used in this study were collected from Formosa cold seep in the SCS during the scientific cruises of “Kexue” in 2017 and 2018 (Figure 1A). To obtain the seawater of animal community (WAC), a syringe-like sampler was used with the aid of a remote operated vehicle (ROV), and seawater closely approaching the seepage was successfully achieved. The seawater closely above the seepage (WCAS) was collected from around two meters above the seepage using a water sampler equipped with ROV. The seawater far above (220 m, 120 m, and 60 m) the seepage (WFAS) was collected with an independent water sampler from the depth of 900 m, 1000 m, and 1060 m. Approximately 3 L seawater from each sample was filtered with 0.22 μm sterile filter membranes. The membranes were stored at −80 °C and used for DNA extraction. Sediments (SA1 and SA2) covered with the animal community were collected by a television grab. To avoid contamination, the top sediment (about one centimeter) was eliminated. Black reduced sediments away from the center (SBa and SBb) were collected with a pushcore equipment and divided into different layers by every two centimeters. The cores were washed with sterile water and kept closed before sampling. After sampling, the cores were taken on board in the closed state. Once on board, the core was opened, and the layer of seawater above the sediment sample inside the core was carefully removed with a syringe. The sample inside the core was pushed up from the bottom of the core, and as the sample emerged at the top end of the core, the subsample was taken for every two centimeters, which was considered one layer. To avoid contamination, the subsample was taken from the center region of each layer. The samples were stored at −80 °C until use. Animal samples, including *Bathymodiolus platifrons* and *Shinkaia crosnieri*, were also collected with a television grab. Once aboard, the specimens were immediately washed thoroughly with sterile seawater, and the gills were taken and stored at −80 °C.

### 2.2. Chemical Analysis

The concentration of CH_4_ in the seawater was detected by a CONTROS HydroC CH_4_ sensor (Kongsberg Gruppen, Norway). Hydrogen sulfide (H_2_S) was measured with the methylene blue method [23] as soon as the water samples were onboard. Dissolved CH_4_ and H_2_S in the surficial reduced sediments were analyzed with in situ Raman spectra as reported previously [24].

### 2.3. NGS Amplicon and Pacbio Amplicon Sequencing

(i) NGS library preparation and sequencing. Total genome DNA was extracted from the samples using the cetyltrimethylammonium bromide and sodium dodecyl sulfate method [25]. DNA concentration and purity were monitored by 1% (*w/v*) agarose gel electrophoresis. DNA concentration was also assessed using Qubit 2.0 Fluorometer (Thermo Scientific, Waltham, USA). The V3-V4 regions of 16S rRNA gene were amplified using universal prokaryotic primers with barcodes as reported previously [26]. Briefly, the universal primer set was 341F (5′-CCTAYGGGRBGCASCAG-3′) and 806R (5′-GGACTACNNGGGTATCTAAT-3′). The PCR reaction was carried out in a 30 μL reaction volume with 15 μL Phusion High-Fidelity PCR Master Mix (New England Biolabs, Ipswich, USA), 0.3 μM forward and reverse primers, and 10 ng template DNA. Sequencing libraries were generated using Ion Plus Fragment Library Kit (Thermo Scientific, Waltham, MA, USA) or TruSeq^®^ DNA PCR-Free Sample Preparation Kit (Illumina, San Diego, CA, USA) following the manufacturer’s recommendations. The library quality was assessed with Qubit 2.0 Fluorometer. The libraries were sequenced using an Ion S5TM XL platform or an Illumina Hiseq2500 platform, and 600 bp single-end reads or 250 bp paired-end reads were generated.

(ii) Pacbio library preparation and sequencing. Nearly complete 16S rRNA genes were amplified using primers 27F/1492R [27]. The PCR reaction was carried out in a 50 μL reaction volume with TransGen High-Fidelity PCR SuperMix (TransGen Biotech, Beijing, China), 0.2 μM forward and reverse primers, and 5 ng template DNA. Thermal cycling consisted of denaturation at 95 °C for 2 min, followed by 35 cycles of 95 °C for 30 s, 60 °C for 45 s, and 72 °C for 90 s, and finally 72 °C for 10 min. Sequencing libraries were generated using SMRTbell TM Template Prep Kit (PacBio, Menlo Park, CA, USA) following manufacturer’s recommendation. The library quality was assessed with Qubit 2.0 Fluorometer and FEMTO Pulse system (Agilent Technologies, Santa Clara, CA, USA). The libraries were sequenced on the PacBio Sequel platform.

### 2.4. Amplicon Data Analysis

(i) Data split. Single-end reads or paired-end reads were assigned to samples based on their unique barcode and truncated by cutting off the barcode and primer sequence. For the Pacbio platform, raw sequences were initially processed through the PacBio SMRT portal. Sequences were filtered for a minimum of three passes and a minimum predicted accuracy of 90% (minfullpass = 3, minPredictedAccuacy = 0.9), which is defined as the threshold below which a circular consensus sequence is considered as noise. The files generated by the PacBio platform were then used for amplicon size trimming to remove sequences outside the expected amplicon size (minLength 1340 bp, maxLength 1640 bp). The reads were assigned to samples based on their unique barcode and truncated by cutting off the barcode and primer sequence.

(ii) Sequence assembly. Paired-end reads were merged using FLASH (v.1.2.7, http://ccb.jhu.edu/software/FLASH/) [28], a very fast and accurate analysis tool designed to merge paired-end reads when at least some of the reads overlap the read generated from the opposite end of the same DNA fragment, and the splicing sequences were called raw tags.

(iii) Data filtration. Quality filtering of the raw reads/tags was performed under specific filtering conditions to obtain high-quality clean reads according to the Cutadapt (v.1.9.1) [29] quality controlled process.

(iv) Chimera removal. The reads were compared with the reference database (Silva database, https://www.arb-silva.de/) [30] using the UCHIME algorithm (http://www.drive5.com/usearch/manual/uchime_algo.html) to detect chimera sequences, which were then removed [31]. The resulting reads were the clean reads.

(v) Operational taxonomic unit (OTU) production and species annotation. Sequence analysis was performed with Uparse v.7.0.1001 (http://drive5.com/uparse/) [32]. Sequences with ≥97% similarity were assigned to the same OTU. Representative sequence for each OTU was screened for further annotation. For each representative sequence, Silva Database [30] was used based on Mothur [33] algorithm to annotate taxonomic information.

(vi) Alpha diversity. Alpha diversity was applied to analyze the complexity of species diversity for a sample through indices, including Observed species, Shannon, Simpson, and Good’s coverage. All these indices in our samples were calculated with QIIME (Version1.9.1) [34] and displayed with R project Vegan package (v.2.5.3) (https://cran.r-project.org/).

(vii) Beta diversity. In beta diversity analysis, nonmetric multidimensional scaling (NMDS) analysis based on weighted UniFrac distances was performed to visualize broad trends of similarities and differences of related samples. Unweighted pair group method using arithmetic average (UPGMA) clustering analysis was performed with the Bray–Curtis distance. Discrimination of microbial community structures of different groups was performed with analysis of similarities (ANOSIM) using PRIMER 6 [35].

### 2.5. Binning Based on Metagenome Data

(i) DNA extraction and library preparation. In total, 1 μg DNA per sample was used as input material for DNA sample preparation. Sequencing libraries were generated using NEBNext^®^ Ultra™ DNA Library Prep Kit for Illumina (New England Biolabs, Ipswich, MA, USA) following the manufacturer’s recommendations and index codes were added to attribute sequences to each sample. Briefly, the DNA sample was fragmented by sonication to a size of 350 bp, and the fragments were end-polished, A-tailed, and ligated with the full-length adaptor for Illumina sequencing. The PCR products were purified with an AMPure XP system (Beckman Coulter, CA, USA), and the libraries were analyzed for size distribution using Agilent2100 Bioanalyzer (Agilent Technologies, Santa Clara, CA, USA) and quantified using real-time PCR (Thermo Scientific, Waltham, MA, USA) [36].

(ii) Sequencing. The clustering of the index-coded samples was performed on a cBot Cluster Generation System (Illumina, San Diego, CA, USA) according to the manufacturer’s instructions. After cluster generation, the library preparations were sequenced on an Illumina HiSeq platform and paired-end reads were generated.

(iii) Clean reads filtering. Quality trimming is an essential step to generate high confidence of variant calling. Raw reads can be processed to get high quality clean reads according to three stringent filtering standards: (1) removing reads with  ≥10% unidentified nucleotides (N); (2) removing reads with  >50% bases having phred quality scores of ≤20; (3) removing reads aligned to the barcode adapter.

(iv) Assembly. The Illumina sequence data of each sample were assembled individually using Megahit [37] stepping over a k-mer range of 21 to 99 to generate sample-derived assembly. Overall de novo assembly statistics were evaluated as a combination of percent paired or singleton reads realigning to the assembly using BWA [38]. The unmapped reads of each sample were pooled to re-assembly using Megahit [37] to generate mixed assembly. Sample-derived assembly and mixed assembly were combined to obtain the final assembly for further analysis.

(v) Chimerism correction. Only contigs larger than 1500 bp were retained. Reads were mapped back to the final contig set using Bowtie (v.2.2.5) [39]. The contig coverage was calculated using bedtools (v.2.29.0) [40] based on the alignment information, using 3000–5999bp sliding windows. The contigs were subsequently broken into separate regions if there were significant read coverage deviations from the average (excluding gaps). For each library, if m1 and m2 are the mean read coverage depth for two adjacent windows (W1 and W2), the break point is between W1 and W2 where: abs(m1-m2)/min (m1,m2) ≥ 0.75 [41].

(vi) Binning. After the correction, MetaBAT (v.2.10.2) [42] was employed to bin the contigs (>1.5 kb) into clusters on the basis of sequence composition and coverage. Single-copy marker gene analysis was performed using CheckM (v.1.0.7) [43] to assess the quality (completeness and contamination) of all bins.

(vii) Annotation. The genes of high quality bins (completeness ≥75% and contamination ≤10%) were compared to non-redundant database (https://www.ncbi.nlm.nih.gov/) using DIAMOND (v.0.9.24) [44], and the top hit, length, and percentage identity were recorded. This allowed us to predict the most likely genus for each contig within each bin.

### 2.6. Phylogenetic Analysis

The phylogenetic tree was constructed using Mega 7.0 [45]. The maximum-likelihood (ML) tree was constructed using nearest-neighbor-interchange heuristic methods, Kimura two-parameter model, uniform rates and complete deletion options. The neighbor-joining (NJ) tree was constructed using Kimura two-parameter model, transitions+transversions, uniform rates and complete deletion options. The maximum-parsimony (MP) tree was constructed using the subtree-pruning-regrafting (SPR) search method and complete deletion option. The robustness of the phylogenetic trees was confirmed by bootstrap analysis based on 1000 replications.

### 2.7. Data Availability

Representative OTU sequences were deposited in GenBank under the accession numbers MT052679–MT052683. NGS amplicon and Pacbio amplicon sequencing data were deposited in the Short Reads Archive (National Center for Biotechnology Information, Bethesda, MD, USA) under the accession number PRJNA606380. The genome assembly of *Sulfurovum* sp. was deposited in GenBank under the accession number JAACKB000000000.

## 3. Results

### 3.1. Physicochemical Characteristics of Sampling Sites

The length and width of the cold seep are approximately 100 m. The center of the seep rose like a hill that harbored a large number of animals dominated by *B. platifrons* and *S. crosnieri* (Figure 1B). There were many carbonate rocks distributed in the center area of the seep. Small bubbles visible to the naked eye floated up from the animal community in the center of the seep. The concentration of CH_4_ was high in the seawater of the animal community (WAC) (>12 μM) and the seawater closely above the seepage (WCAS) (3–9 μM), but very low in the seawater far above the seepage (WFAS) (<1 μM). The concentration of H_2_S was high in WAC (~20 μM), but undetected in WCAS and WFAS. Of the sediment samples, SA1 and SA2 were sediments from the areas of animal community; SBa and SBb were black reduced sediments from the areas away from the seepage and the animal community (Figure 1C), in which high concentrations of CH_4_ (4.7 mM) and H_2_S (10 mM) were detected. The sample informations are listed in Supplementary Table S1.

### 3.2. Sequencing Data and Alpha Diversity

All samples were subjected to sequencing analysis. Thirty-six NGS amplicon libraries and 10 Pacbio amplicon libraries were constructed. A total of 2436372 effective tags of 16S rDNA (average length of ~400 bp) spanning the variable regions of V3 and V4 were recovered from the NGS amplicon libraries, and 96638 subreads of 16S rDNA with an average length of ~1500 bp were obtained from the Pacbio amplicon libraries. Good’s coverage of each sample was estimated, and the values of all samples in NGS amplicon libraries were above 99.5%, indicating that all libraries represented well the microbial communities (Supplementary Table S2). The Good’s coverage values in the Pacbio amplicon libraries were also good enough (>90.0%) to assess the microbial diversity of the samples, with the exception of SBB12-14 (83.8%) and SBB16-18 (77.0%), which, as shown below, had more complex communities (Supplementary Table S3). The Shannon–Wiener diversity indices indicated that the microbial communities in sediment samples (7.20 ± 1.96) were more sophisticated than that in seawater (5.82 ± 1.16) and animal samples (2.82 ± 0.76) (Supplementary Table S2).

### 3.3. Microbial Communities in the Seawater Samples

**Figure 2:**
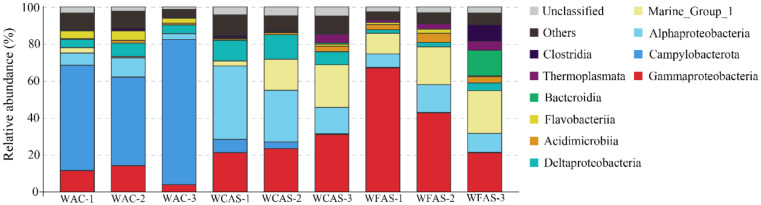
The diversity and distribution of microbial sequence tags in the libraries of seawater samples. Libraries of the seawater of animal community (WAC) are indicated by WAC-1, WAC-2, and WAC-3; libraries of the seawater closely above the seepage (WCAS) are indicated by WCAS-1, WCAS-2, and WCAS-3; libraries of the seawater far above (60 m, 120 m, and 220 m) the seepage (WFAS) are indicated by WFAS-1, WFAS-2 and WFAS-3. The sequence tags were generated with the next-generation sequencing (NGS) platform and classified at the phylum/class level. Each color represents the percentage of the taxon in the total assemblage. The top 10 groups in each sample are labeled, and other groups are integrated as “Others”.

NMDS analysis showed that the WAC samples were separated from the WCAS and WFAS samples in the plot, implying that the microbial communities in WAC were different from that in WCAS and WFAS (Supplementary Figure S1). ANOSIM analysis indicated that the R value between WAC and WFAS was 1.0, and the R value between WAC and WCAS was 0.93, implying a large difference in the communities between WAC and WFAS and between WAC and WCAS. In WAC, Campylobacterota was dominant, with an average percentage of 61.1 ± 15.3%, which was followed in abundance by Gammaproteobacteria (9.9 ± 5.1%), Alphaproteobacteria (6.7 ± 3.7%), and Deltaproteobacteria (5.4 ± 1.4%) (Figure 2). The Pacbio amplicon data also indicated that in WAC, Campylobacterota was dominant in the microbial community (Supplementary Figure S2). In WCAS, Alphaproteobacteria was most abundant, with an average percentage of 27.3 ± 12.7%, which was followed in abundance by Gammaproteobacteria (25.2 ± 5.1%), Marine Group I (14.2 ± 10.4%), and Deltaproteobacteria (10.5 ± 3.2%) (Figure 2). In WFAS, Gammaproteobacteria was the dominant class (43.6 ± 22.9%) and followed by Marine Group I (18.2 ± 6.2%) and Alphaproteobacteria (10.8 ± 3.9%) (Figure 2).

**Figure 3:**
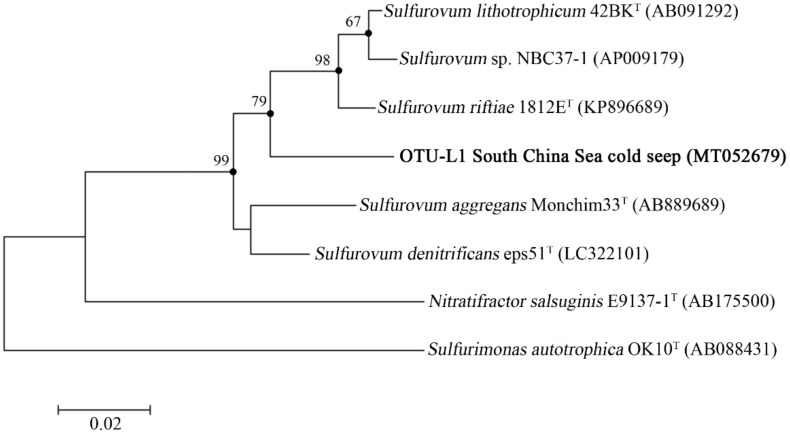
The maximum-likelihood phylogenetic tree based on nearly complete 16S rRNA gene sequences (1388 bp) showing the positions of operational taxonomic unit—L1 (OUT-L1) from this study, members of *Sulfurovum*, and representatives of some other related taxa. GenBank accession numbers are shown in parenthesis. Filled circles indicate that the corresponding nodes were also recovered in the trees generated with the neighbor-joining and maximum-parsimony algorithms. Only bootstrap values (expressed as percentages of 1000 replications) equal or greater than 50% are shown at the branching points. *Sulfurimonas autotrophica* OK10^T^ (GenBank accession number, AB088431) was used as an outgroup. Scale bar, 0.02 substitutions per nucleotide position.

In OTU level, NGS data showed that most Campylobacterota tags (79.6%) in WAC were classified into OTU-S1, which belonged to the genus *Sulfurovum*. The Pacbio amplicon data also indicated that in WAC, nearly all Campylobacterota subreads belonged to this genus, and most *Sulfurovum* subreads (86.7%) were classified into OTU-L1. Sequence analysis indicated that in their shared region, OTU-S1 differed from OTU-L1 only in 1 base. Sequence analysis with the Basic Local Alignment Search Tool for nucleotide (BLASTn) showed that OTU-L1 was very close to the uncultured clones from whale falls, methane seep, and hydrothermal vents, indicating that close relatives of OTU-L1 were widespread in reduced marine environments (Supplementary Table S4). Phylogenetic analysis showed that OTU-L1 was clustered with *Sulfurovum* sp. NBC37-1, *Sulfurovum lithotrophicum*, *Sulfurovum denitrificans*, *Sulfurovum riftiae*, and *Sulfurovum aggregans* (Figure 3), indicating that the uncultured strains in the seawater samples represented by OTU-L1 belonged to *Sulfurovum* genus. Sequence analysis demonstrated that the identities between OTU-L1 and its close type strains *S. lithotrophicum*, *S. denitrificans*, *S. riftiae*, and *S. aggregans* were 96.0%, 95.6%, 95.5%, and 94.9%, respectively, each of which was well below the threshold of 97% that has been suggested for bacterial species delineation, suggesting that the uncultured strain of the seawater represented by OTU-L1 was a potentially new species of the genus *Sulfurovum*.

### 3.4. Microbial Communities in the Sediment Samples

**Figure 4:**
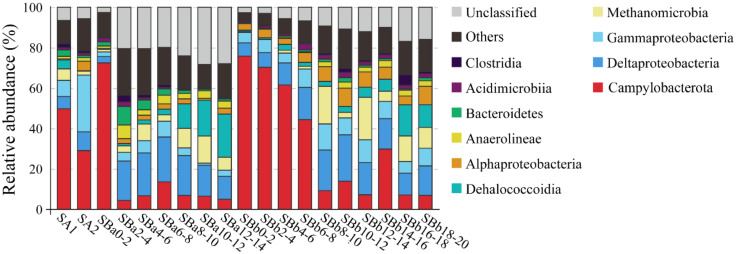
The diversity and distribution of microbial sequence tags in the libraries of sediment samples. SA1 and SA2 represent the libraries of the sediments covered with the animal community; SBa and SBb represent the libraries of the sediments away from the center, with the numbers after SBa and SBb indicating the depth (cm). The sequence tags were generated with the next-generation sequencing (NGS) platform and classified at the phylum/class level. Each color represents the percentage of the taxon in the total assemblage. The top 10 groups in each sample are labeled, and other groups are integrated as “Others”.

In both SA1 and SA2, which were associated with animal community, Campylobacterota was abundant (49.7 and 29.0%, respectively) (Figure 4). Most Campylobacterota tags (76.7 and 85.9% in SA1 and SA2, respectively) were classified into OTU-S1. The other abundant groups included Gammaproteobacteria, Deltaproteobacteria, and Methanomicrobia (>2%) (Figure 4). 

SBa was 14 cm long and equally divided into 7 layers named SBa0-2 (meaning 0–2 cm, the same for below), SBa2-4, SBa4-6, SBa6-8, SBa8-10, SBa10-12, and SBa12-14. Campylobacterota was overwhelmingly dominant in the top layer of SBa0-2 (73.9%), but was low in abundance in the deeper layers of 2 cm to 14 cm (5.4–15.5%) (Figure 4). On the contrary, Deltaproteobacteria, Methanomicrobia, and Dehalococcoidia were rare in the top layer but abundant in the subsurface. The abundance of Deltaproteobacteria in the first layer was 3.1%, which rose up to the highest percentage of 22.2% in the fourth layer, and then declined to 11.3% in the bottom layer (Figure 4). The abundance of Methanomicrobia was 1.3% in the top layer and increased in the subsurface, reaching up to the highest level of 13.4% in SBa10-12 (Figure 4). The abundance of Dehalococcoidia was 0.9% in the top layer, and ascended with depth, reaching up to 21.4% in the bottom layer of SBa12-14 (Figure 4).

SBb was 20 cm long and equally divided into 10 layers named SBb0-2 (meaning 0–2 cm, the same for below), SBb2-4, SBb4-6, SBb6-8, SBb8-10, SBb10-12, SBb12-14, SBb14-16, SBb16-18, and SBb18-20. A microbial distribution tendency similar to that of SBa occurred in SBb, but with some differences. In the top four layer (SBb0-2, SBb2-4, SBb4-6, and SBb6-8) and the eighth layer (SBb14-16), Campylobacterota was the most abundant (29.8–77.8%), while in the other layers, the percentages of Campylobacterota were only 6.9–13.9% (Figure 4). The abundance of Deltaproteobacteria was 6.5% in the top layer (SBb0-2) and increased with depth, reaching up to 23.0% in SBb10-12, and then declined (Figure 4). The abundances of Methanomicrobia were low (0.2–1.5%) in the top four layers, which rose up to 18.6% and 21.0% in SBb8-10 and SBb12-14, respectively, and then decreased to 2.8% and 12.6% in the other layers (Figure 4). The abundances of Dehalococcoidia in the top eight layers (0–16 cm) were low (0.7 and 5.9%), but rose up to 15.5% and 11.3% in the ninth and tenth layer, respectively (Figure 4). Cluster analysis indicated that all surface sediment samples were clustered into a single group, while the middle and bottom layers of samples were clustered into another group (Supplementary Figure S3). These results indicated that the communities of surface sediments were different from the communities of the middle and bottom sediments.

**Figure 5:**
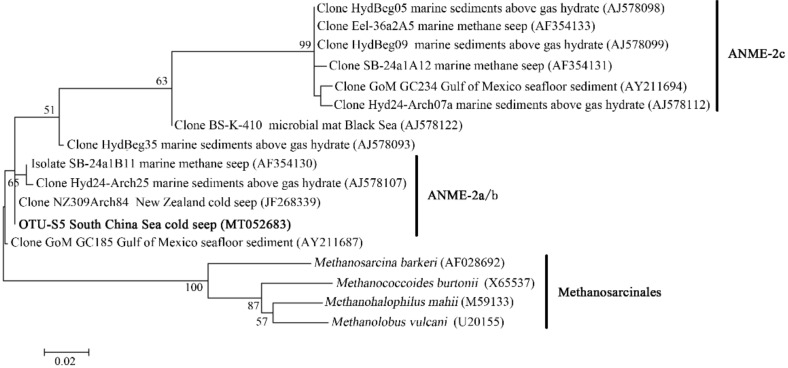
The maximum-likelihood phylogenetic tree based on 16S rRNA gene sequences (391 bp) showing the position of OTU-S5 from this study. Close sequences from National Center for Biotechnology Information (NCBI) database were selected to construct the tree. GenBank accession numbers are shown in parenthesis. Only bootstrap values (expressed as percentages of 1000 replications) equal or greater than 50% are shown at the branching points. Scale bar, 0.02 substitutions per nucleotide position.

**Figure 6:**
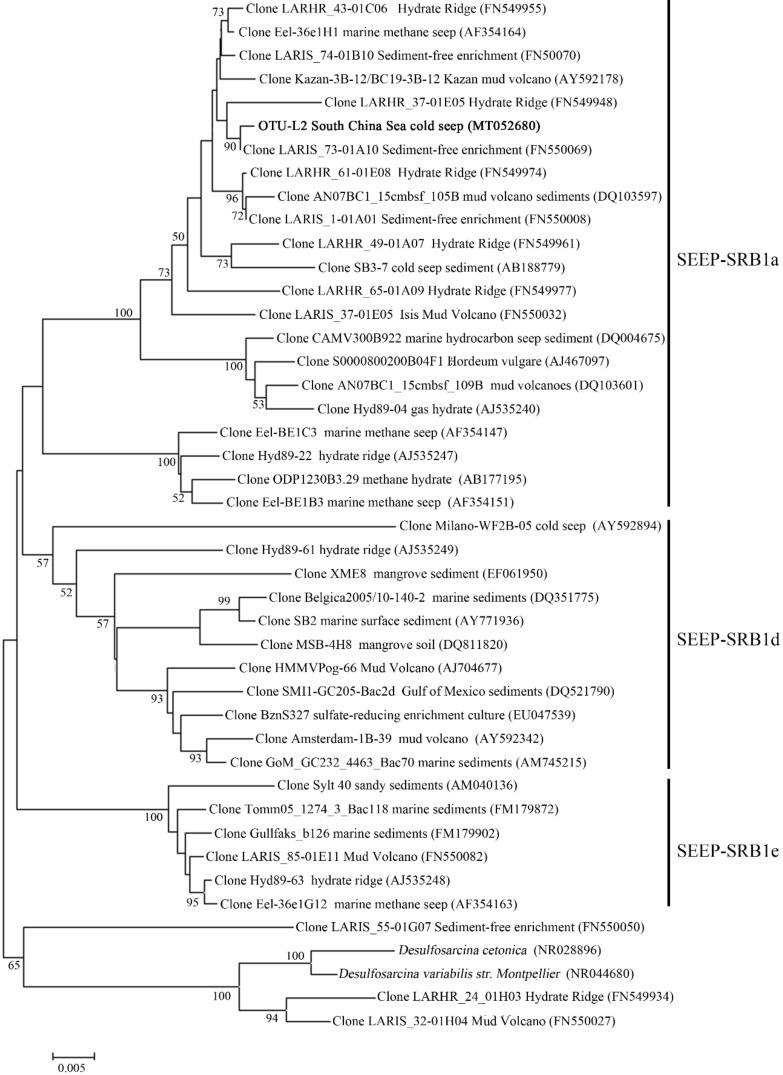
The maximum-likelihood phylogenetic tree based on 16S rRNA gene sequences (1188 bp) showing the position of OTU-L2 from this study. GenBank accession numbers are shown in parenthesis. Sequences within seep sulfate-reducing bacteria—1 (SEEP-SRB1) were selected according to Schreiber et al. 2010. Only bootstrap values (expressed as percentages of 1000 replications) equal or greater than 50% are shown at the branching points. Scale bar, 0.005 substitutions per nucleotide position.

In the OTU level, most Campylobacterota tags (85.5%) in SBa and SBb were classified as OTU-S1, which indicated that OTU-S1 was dominant in the surface sediment communities. Consistent with the NGS amplicon sequencing results, the Pacbio amplicon sequencing data showed that Campylobacterota was dominant in SBb, most of which were classified as OTU-L1 that accounted for 98.1% and 96.0% in SBb0-2 and SBbB4-6, respectively (Supplementary Figure S2). These results indicated that *Sulfurovum* was dominant in the surface sediments. In the middle and bottom layers, Methanomicrobia was abundant, and the most abundant OTU was OTU-S5. Phylogenetic analysis indicated that OTU-S5 was clustered in the ANME-2 a/b group (Figure 5), suggesting an involvement in methane oxidation. Deltaproteobacteria was also abundant in the middle and bottom layers, and the most abundant OTU was OTU-S7. In the Pacbio amplicon sequencing data, OTU-L2 was highly similar to OTU-S7 (only 4 bp difference) and was used to perform phylogenetic analysis, which showed that OTU-L2 was clustered within the SEEP-SRB1a group (Figure 6) that is usually viewed as a partner of ANME2 [46].

### 3.5. Microbial Communities of S. crosnieri and B. platifrons

**Figure 7:**
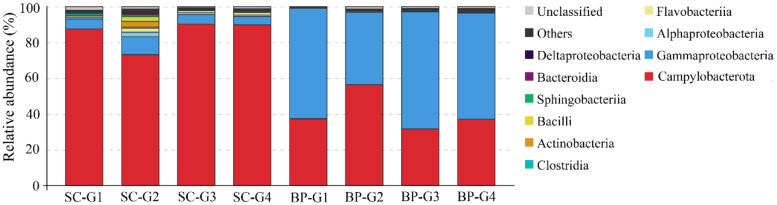
Diversity and distribution of microbial sequence tags in animal-associated libraries. SC-G1 SC-G2, SC-G3, and SC-G4 represent the libraries of the gills of *Shinkaia crosnieri*; BP-G1, BP-G2, BP-G3, BP-G4 represent the libraries of the gills of *Bathymodiolus platifrons*. The sequence tags were generated with the next-generation sequencing platform and classified at the phylum/class level. Each color represents the percentage of the taxon in the total assemblage. The top 10 groups in each sample are labeled, and other groups are integrated as “Others”.

In the gills of *S. crosnieri*, Campylobacterota dominated the microbial communities, with an average percentage of 85.2 ± 8.1% (Figure 7). The next abundant group was Gammaproteobacteria (6.4 ± 2.4%) (Figure 7). The other groups were lower than 2%. Most Campylobacterota tags (61.5%) was classified into OTU-S1, indicating that *Sulfurovum* was abundant in the gills of *S. crosnieri*.

**Figure 8:**
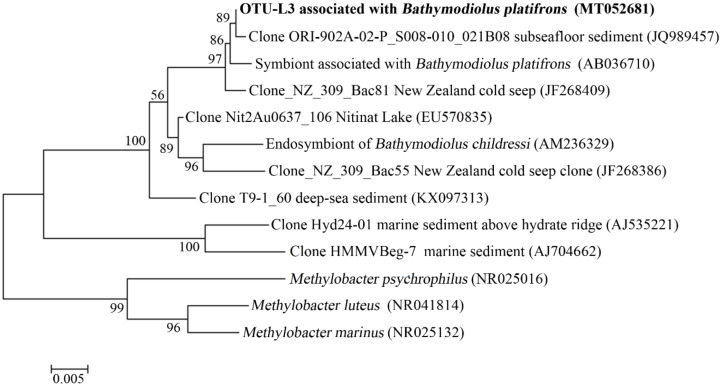
The maximum-likelihood phylogenetic tree based on 16S rRNA gene sequences showing the position of OTU-L3 from this study. Close sequences from NCBI database were selected to construct the tree. GenBank accession numbers are shown in parenthesis. Only bootstrap values (expressed as percentages of 1000 replications) equal or greater than 50% are shown at branching points. Scale bar, 0.005 substitutions per nucleotide position.

**Figure 9:**
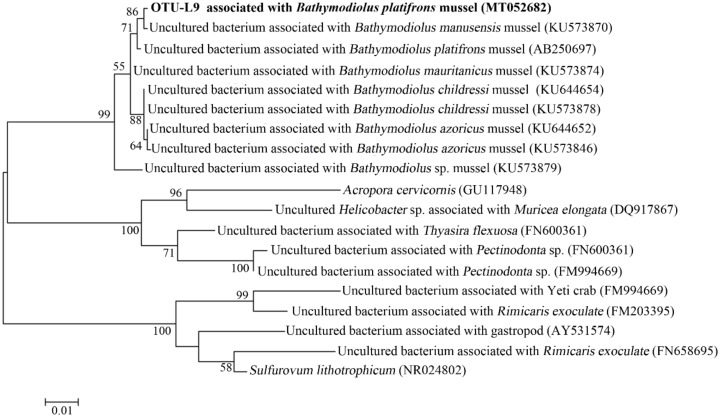
The maximum-likelihood phylogenetic tree based on nearly complete 16S rRNA gene sequences (1417 bp) showing the position of OTU-L9 from this study. Close sequences from NCBI database were selected to construct the tree. GenBank accession numbers are shown in parenthesis. Only bootstrap values (expressed as percentages of 1000 replications) equal or greater than 50% are shown at the branching points. Scale bar, 0.01 substitutions per nucleotide position.

In the gills of *B. platifrons*, Gammaproteobacteria and Campylobacterota dominated the microbial communities, with average percentages of 56.8 ± 11.1% and 40.6% ± 10.8 %, respectively (Figure 7). The other groups were scarce (<1% in percentage). In OTU level, most Gammaproteobacteria tags were classified into OTU-S5 and OTU-S958, accounting for 33.8 ± 7.4% and 21.3 ± 4.3% of the gill communities, respectively. The majority of the Campylobacterota tags (87.2%) were classified into OTU-S4. In the Pacbio amplicon sequencing data, Gammaproteobacteria was dominant with an overwhelming percentage of 89.3 ± 2.3%, while Campylobacterota was 10.0 ± 2.0% in percentage (Supplementary Figure S4). In OTU level, nearly all Gammaproteobacteria subreads (96.8%) were classified into OTU-L3. OTU-L3 was completely identical to OTU-S5 in their shared region, and differed from OTU-S958 in five bases. Phylogenetic analysis indicated that OTU-L3 was clustered together with methane-oxidizing bacteria found in both deep-sea animals, such as mussels, and environmental samples, such as cold seep sediments (Figure 8). The majority of Campylobacterota tags (90.0%) were classified into OTU-L9, which was phylogenetically clustered into the group of bacteria that were specifically associated with deep-sea mussels (Figure 9) and represented a new family [21].

### 3.6. Genome Analysis of Sulfurovum sp.

Based on the data of metagenome, a draft genome of *Sulfurovum* sp. was obtained by binning. The estimated size of the genome is 2.12M bp, with 84.0% completeness and 2.1% contamination (Supplementary Table S5). A total of 4027 contigs were assembled and 1070 genes were predicted (Supplementary Table S5). Kyoto Encyclopedia of Genes and Genomes (KEGG) analysis identified the gene associated with carbon fixation, such as adenosine triphosphate citrate synthase, which is a key gene of the reductive tricarboxylic acid (rTCA) pathway, and the genes of the sulfur oxidizing (Sox) pathway, including SoxYZ and SoxCD, which are key genes of sulfur oxidation (Supplementary Table S6). In addition, nitrate reduction associated gene was found in the genome, suggesting nitrate may be the electron acceptor of *Sulfurovum* sp. (Supplementary Table S6).

## 4. Discussion

Owing to their high accuracy and high-throughput, amplicon sequencing techniques based on NGS platforms have been widely used for assessment of microbial communities in various environments [47,48]. However, the 16S rDNA obtained by NGS are short and cannot be accurately classified in low taxonomic levels [49]. This disadvantage is amplified when NGS is applied to the study of the environments where there are a large number of uncultivable bacteria, such as deep-sea hydrothermal vents and cold seeps. The nearly full-length 16S rDNA amplicon sequencing techniques based on the third generation sequencing platforms, such as Pacbio sequencing platform, are a good complement to the above shortcoming [49]. These new techniques can obtain near complete 16S rDNA, which enables the classification of the microbes into more accurate taxonomic status, and meanwhile the data thus gained will greatly improve the usability of the reference dataset. Moreover, a circular consensus sequence strategy has greatly improved the accuracy of Pacbio sequencing platforms, which, alongside with cost reduction, enables this technique to play an increasingly important role in the prediction of environmental microbial community structures in the future [50]. In addition, since the majority of microorganisms are uncultivable in many environments, metagenomics is an important means to study the function of environmental microbial flora, and the binning technology based on metagenomics allows us to obtain the genome sketches of uncultivable strains, especially some highly abundant strains, which helps to better understand the function of the microbes [51,52]. In this study, using these high through-put techniques, we examined the structure and function of the microbes, especially that with high-abundance, in the Formosa cold seep of the SCS.

### 4.1. Microbial Communities in Cold Seep Seawater

To our knowledge, no reports on the microbial communities in the seawater of deep-sea cold seeps have been documented. In the present study, hydrogen sulfide was found high in the seawater close to the seepage (WAC) in the Formosa cold seep of the SCS, but very low in the seawater 2 to 220 m away from the seepage (WCAS and WFAS). The structures of the microbial communities were also different between WAC and WCAS/WFAS, likely due to the difference of hydrogen sulfide in these areas. In WAC, *Sulfurovum* was found to be the dominant genus. To date, only four *Sulfurovum* species have been isolated, all from deep-sea hydrothermal vents [53,54,55,56], which, like the cold seep in our study, are H_2_S-rich. These observations implied that high-concentrations of H_2_S contributed to the enrichment of *Sulfurovum*. The Pacbio amplicon sequencing technique enabled the obtainment of nearly complete 16S rRNA gene sequence, and subsequent OTU analysis identified the dominant OTU to be OTU-L1, which belonged to *Sulfurovum*. Sequence alignment showed that the identities between OTU-L1 and the four type strains of *Sulfurovum* were less than 97%, suggesting that the main *Sulfurovum* in WAC was a potentially new species. Previous studies indicated that of the four species of *Sulfurovum*, three of them, i.e., *S. denitrificans*, *S. aggregans*, and *S. lithotrophicum*, were microaerobic and could utilize oxygen as an electron acceptor [53,54,55]. Therefore, it is possible that the uncultured *Sulfurovum* represented by OTU-L1 in our study was oxygen-tolerant, since oxygen was detected in WAC.

### 4.2. Microbial Communities Associated with Animals

In the Formosa cold seep of the SCS, *S. crosnieri* and *B. platifrons* were the most abundant benthic macrofauna, especially in the areas close to the seepage. With the samples from these animals, we found that *Sulfurovum* was the most abundant genus in the gills of *S. crosnieri*, and most of the *Sulfurovum* belonged to OTU-L1. In a previous study, abundant *Sulfurovum* was also found in the gills of *S. crosnieri* collected from the hydrothermal vent in Okinawa Trough [57]. These observations suggested a specific association between *Sulfurovum* and the gills of deep sea *S. crosnieri*, which is in line with the report that there might be a specific interaction between deep-sea shrimp and their epibiont bacteria, though the underlying mechanism is unclear [58]. Considering that there existed abundant free-living *Sulfurovum* in the habitat of *S. crosnieri* in the Formosa cold seep, the epibiont *Sulfurovum* in the gill of *S. crosnieri* was likely originated from the ambient environment.

A large number of Gammaproteobacteria and a small amount of Campylobacterota existed in the gills of *B. platifrons*, which was similar to the findings of a previous study [13]. Phylogenetic analysis indicated that most Gammaproteobacteria belonged to the methane-oxidizing bacterial group, while most Campylobacterota belonged to a new family of Bathymodiolinae-associated bacteria, which is unknown in function [21]. OTU-L3, which represented nearly all Gammaproteobacteria subreads, had high identities to the clones from various samples, including those from mussels and cold seep sediments, suggesting a possible horizontal transfer of these bacteria from local environments to animal inhabitants. Conversely, for the main Campylobacterota represented by OTU-L9, its close relatives were almost exclusively associated with mussels, indicating the possibility of vertical transmission.

### 4.3. Microbial Community in Sediments

Based on the analysis of microbial communities in the sediment samples from different sites and depths, we found that there was a high abundance of *Sulfurovum* (OTU-L1) in the surface sediments of the cold seep. A previous report showed that Campylobacterota, including *Sulfurovum*, was abundant in the surface sediments of the same cold seep [59]. The vertical distribution analysis indicated that the depth-dependent distribution of OTU-L1 differed in different sites. In SBa, OTU-L1 was abundant at 0–2 cm, while in SBb, OTU-L1 was abundant at 0–8 cm. Considering that the *Sulfurovum* represented by OTU-L1 may be microaerobic, its distribution was likely affected by oxygen concentration, which may be different in different areas of the sediments, thus accounting for the difference in the depth-dependent abundance of *Sulfurovum* in SBa and SBb. In contrast to *Sulfurovum*, ANME2 and SRB-1a had similar distribution patterns in different sediment samples, and both were abundant in the middle and bottom layers of SBa and SBb. Previous reports indicated that these two groups of organisms cooperated to complete anaerobic oxidation of methane [46]. Therefore, it is likely that ANME2 and SRB-1a may play an important role in methane oxidation in the Formosa cold seep of the SCS.

### 4.4. Genome Analysis of the Sulfurovum sp.

Using the binning technique based on metagenomic data, we succeeded in obtaining a draft genome of the *Sulfurovum* sp. from the surface sediment sample. Since there was a high-abundance of OTU-L1 in this sample, this genome may represent the strain belonging to OTU-L1. In the genome sketch, the genes related to the rTCA pathway, Sox pathway, and nitrate-reduction pathway were discovered, suggesting that these pathways likely function in carbon fixation, oxidizing reduced sulfur to provide energy, and electron transfer, respectively. These results were consistent with the reported metabolic pathways of an isolate of *Sulfurovum* [60].

## 5. Conclusions

**Figure 10:**
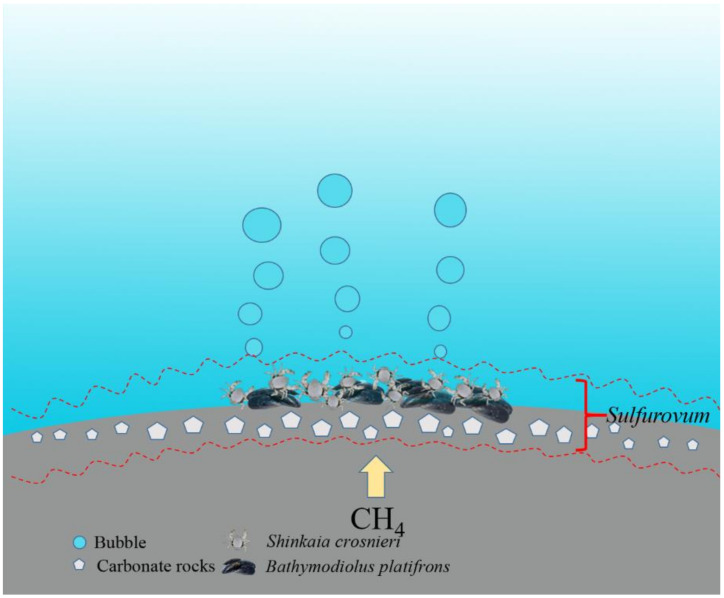
A sketch of *Sulfurovum* distribution in the seawater–sediment interface of Formosa cold seep area.

In conclusion, our study demonstrated that in the Formosa cold seep in South China Sea, *Sulfurovum* was dominant in the sediment–seawater interface samples ranging from water to sediments and inhabitant animals. Furthermore, the dominant *Sulfurovum* was a potentially novel species that may utilize the rTCA pathway, Sox pathway, and nitrate reduction pathways in their metabolism. Based on these observations, we propose a hypothesis as follows (Figure 10): there exists a “*Sulfurovum* layer” in the seawater–sediment interface of Formosa, with boundaries in different areas of the interface fluctuating slightly as a result of variations in oxygen and sulfide hydrogen conditions, and these *Sulfurovum* may play a vital biogeochemical role in the cycling of sulfur, carbon, and nitrogen in the cold seep.

## Supplementary Materials

The following are available online at https://www.mdpi.com/2076-2607/8/5/687/s1.
