# Supplementary material for: High-Throughput Sequencing Reveals a Potentially Novel Sulfurovum Species Dominating the Microbial Communities of the Seawater–Sediment Interface of a Deep-Sea Cold Seep in South China Sea
Source: Microorganisms. 2020 May 8;8(5):687. doi: 10.3390/microorganisms8050687 (PMC7284658; doi:10.3390/microorganisms8050687)
Supplement: Supplementary file 1 [file microorganisms-08-00687-s001.zip › Supplementary data.pdf]

Supplementary data

Table S1. Sample information.

Table S2. Summary of amplicon sequencing information based on the next-generation sequencing platform.

Table S3. Summary of sequencing information based on the Pacbio sequencing platform.

Table S4. Clones from NCBI that are close to OTU-L1. Clones with 98.55% and higher similarities to OTU-L1 are shown.

Accession No.	Identity	isolation source
GQ261780	99.10%	Deep sea sediment associated with whale falls
KX097744	98.97%	Hydrothermal deposits and seafloor sediments across Okinawa trough
GQ357003	98.97%	Methane seep sediment
AM745152	98.97%	Marine sediments
KX097275	98.90%	Hydrothermal deposits and seafloor sediments across Okinawa trough
KX097389	98.83%	Hydrothermal deposits and seafloor sediments across Okinawa trough
GQ356979	98.83%	Methane seep sediment
AY197375	98.76%	Guaymas basin hydrothermal vent sediments
KX097506	98.76%	Hydrothermal deposits and seafloor sediments across Okinawa trough
KF545022	98.76%	Deep-sea sediment
AF420344	98.76%	Guaymas basin hydrothermal vent sediments
KX097364	98.69%	Hydrothermal deposits and seafloor sediments across Okinawa trough
KX097354	98.69%	Hydrothermal deposits and seafloor sediments across Okinawa trough
AY549001	98.69%	Marine environment
KP091181	98.62%	Guaymas basin hydrothermal sediments
AB015531	98.62%	Deep-sea sediments
KM356278	98.55%	Hydrate ridge
KX097729	98.55%	Hydrothermal deposits and seafloor sediments across Okinawa trough
JQ036283	98.55%	Hydrate ridge sediment
JN873954	98.55%	Hydrothermal plumes

Table S5. The genome information of *Sulfurovum* sp..

Genome data	*Sulfurovum* sp.
Estimated size (bp)	2124559
Size (bp)	1998954
Contigs	4027
Gene	1070
GC%	37.59%
Genes with function prediction in Nr database	3096

Table S6. Genes associated with carbon fixation, sulfur oxidation, and nitrate reduction in the *Sulfurovum* genome assembly.

Gene code	Length	E value	Function annotation
Carbon fixation			
0131	207	1e-38	ATP citrate synthase
0810	198	4e-36	ATP citrate synthase
Sulfur oxidation			
0921	300	3e-49	Sulfur oxidation protein SoxYZ
0922	558	5e-67	Sulfur oxidation protein SoxYZ
0923	360	1e-36	Sulfur oxidation protein SoxCD
Nitrate reduction			
0165	123	2.00E-10	Nitrate reductase catalytic subunit

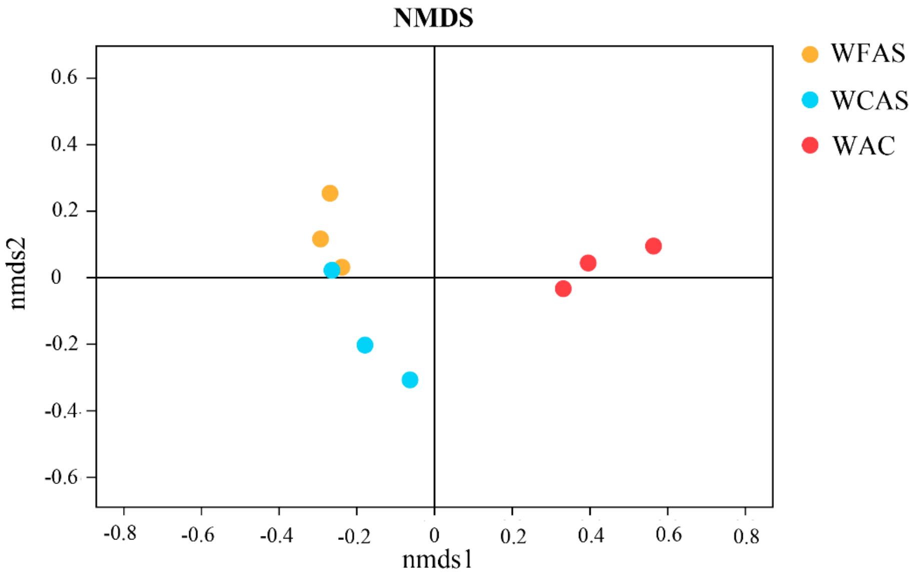

Figure S1. NMDS plot showing the differences of microbial communities in the nine seawater samples.

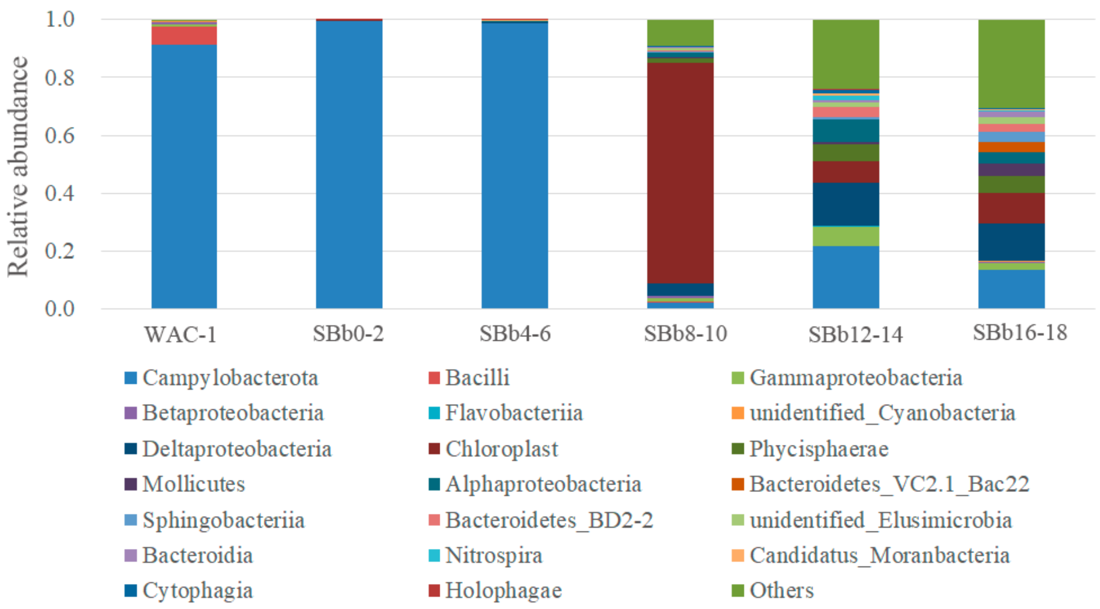

Figure S2. Diversity and distribution of microbial sequence subreads in seawater and sediment libraries. WAC-1 represents the library of the seawater of animal community (WAC); SBb represent the libraries of the sediments away from the center, with the number after SBb indicating the depth (cm). The subreads were generated with the Pacbio sequencing platform and classified at the phylum/class level. Each color represents the percentage of the taxon in the total assemblage. Top 10 groups in each sample are labeled, and other groups are integrated as "Others".

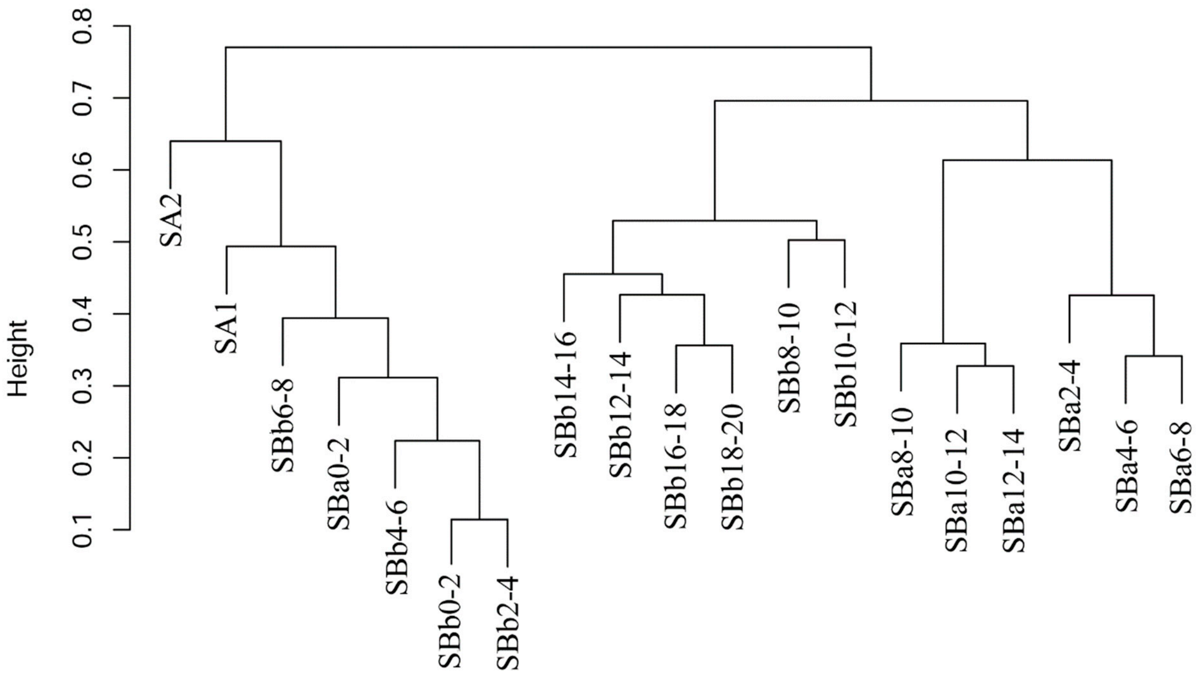

Figure S3. Cluster analysis of sediment samples. The cluster dendrogram was constructed at the class level.

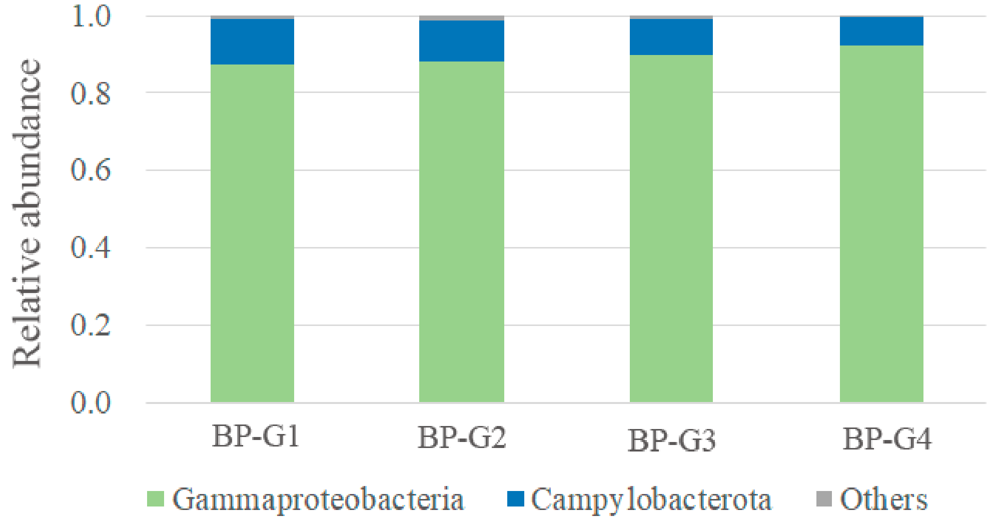

Figure S4. Diversity and distribution of microbial sequence subreads in animal-associated libraries. BP-G1, BP-G2, BP-G3, BP-G4 represent the libraries of the gills of *Bathymodiolus platifrons*. The subreads were generated with the Pacbio sequencing platform and classified at the phylum/class level. Each color represents the percentage of the taxon in the total assemblage. Top 10 groups in each sample are labeled, and other groups were integrated as "Others".
