# Supplementary figures and images for: High-Throughput Sequencing Reveals a Potentially Novel Sulfurovum Species Dominating the Microbial Communities of the Seawater–Sediment Interface of a Deep-Sea Cold Seep in South China Sea
Source: Microorganisms. 2020 May 8;8(5):687. doi: 10.3390/microorganisms8050687 (PMC7284658; doi:10.3390/microorganisms8050687)

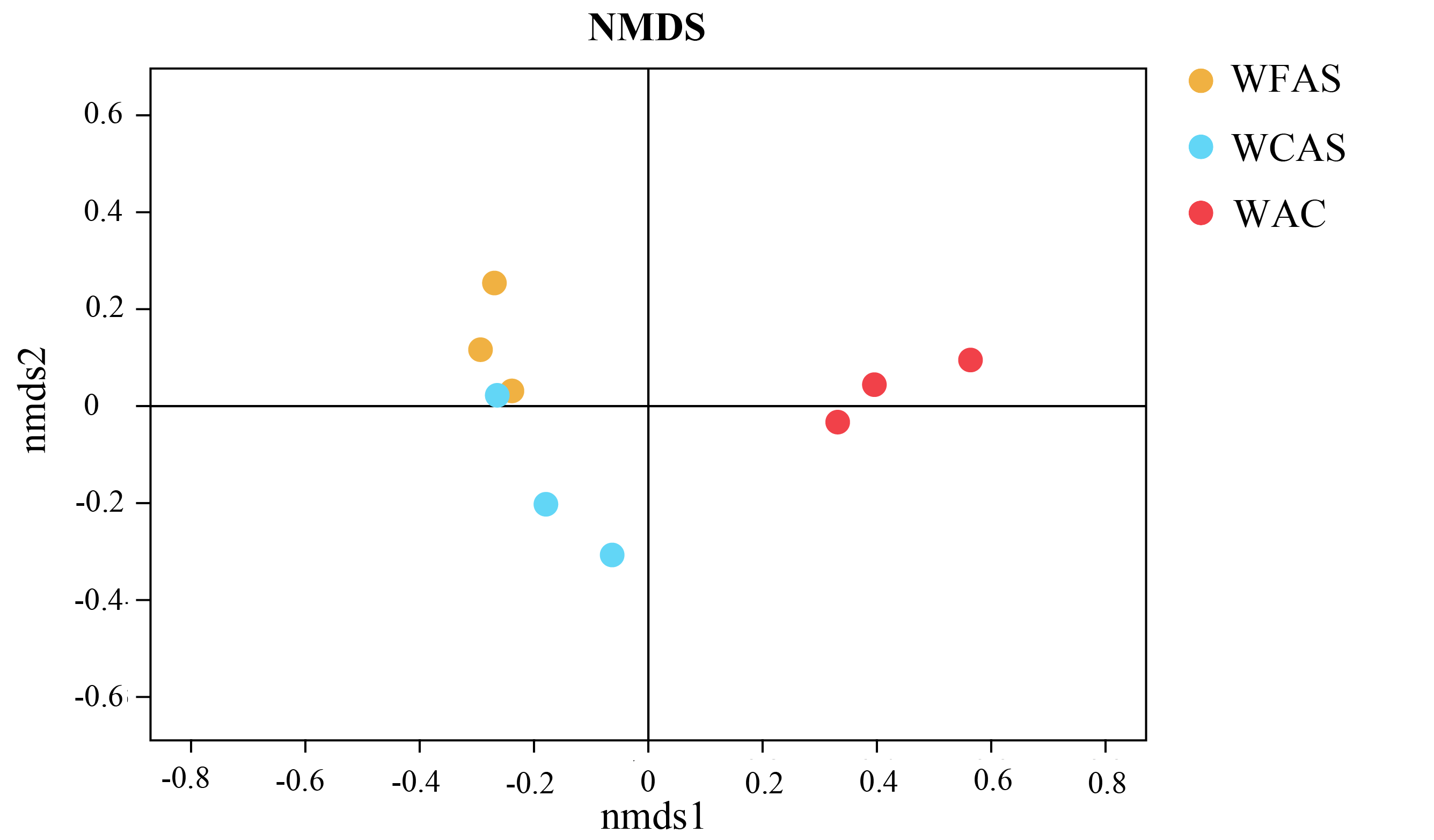

Supplement: Supplementary file 1 [file microorganisms-08-00687-s001.zip › Figure S1.tif]

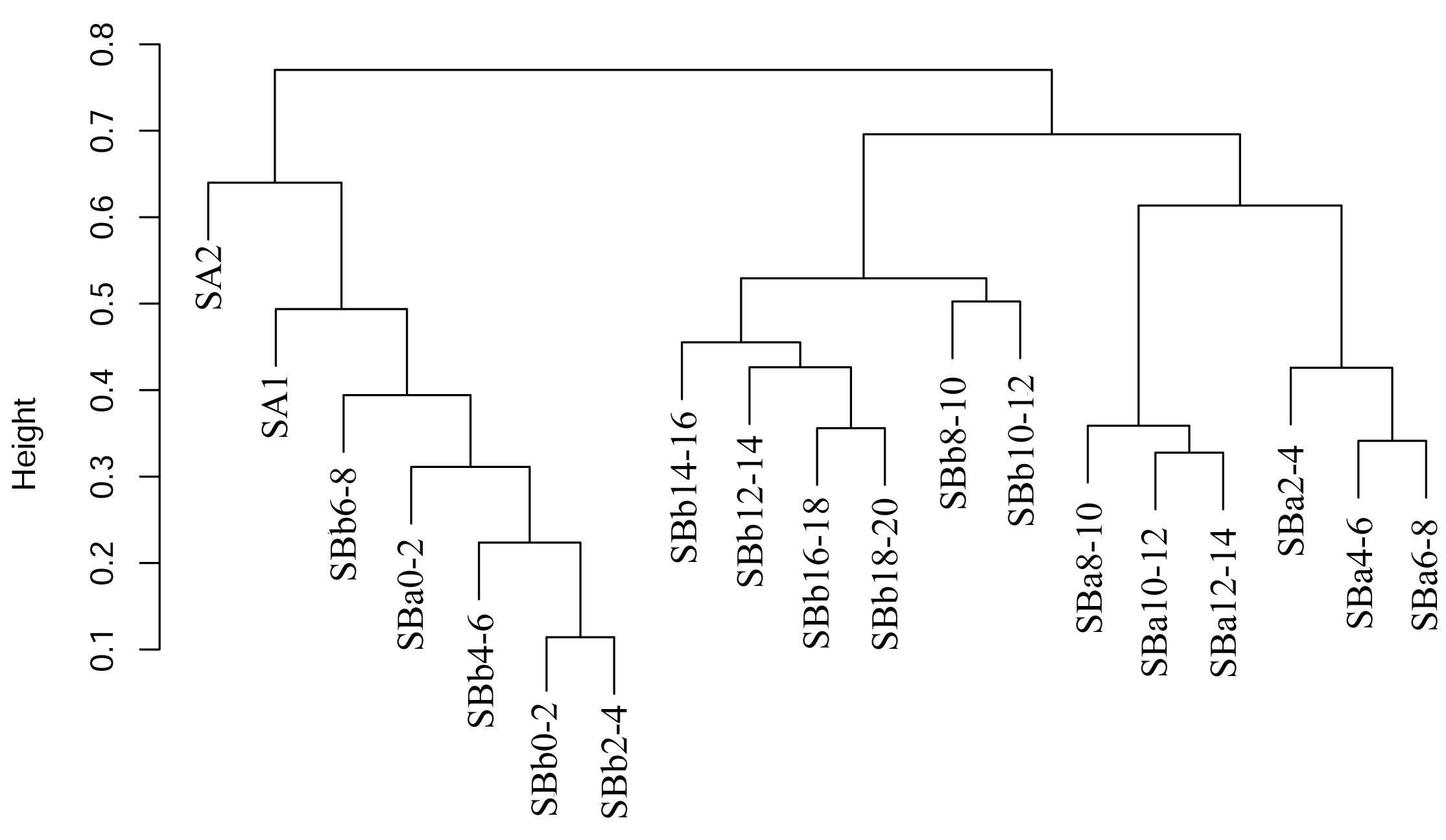

Supplement: Supplementary file 1 [file microorganisms-08-00687-s001.zip › Figure S2.tif]

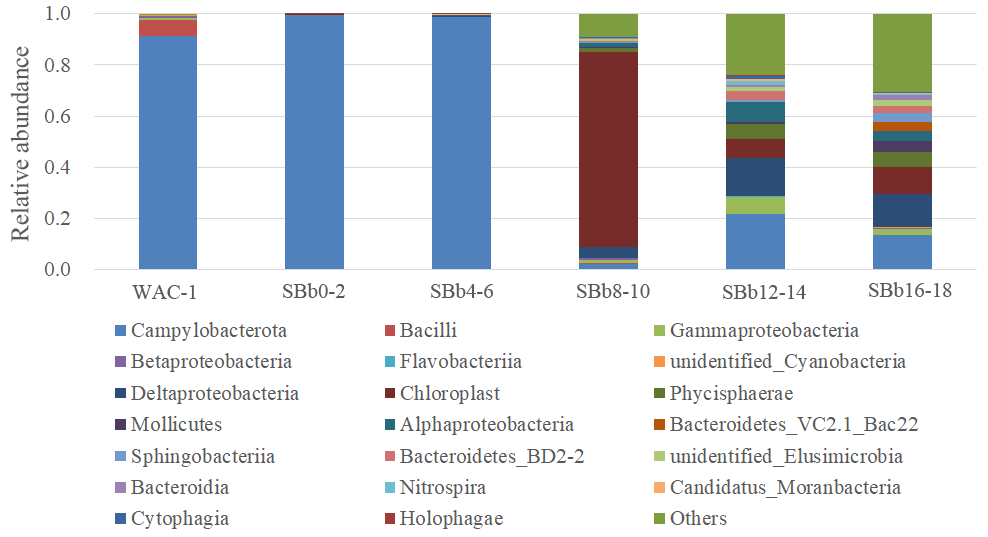

Supplement: Supplementary file 1 [file microorganisms-08-00687-s001.zip › Figure S3.tif]

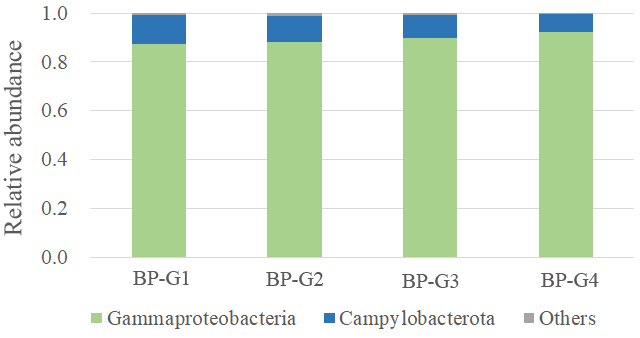

Supplement: Supplementary file 1 [file microorganisms-08-00687-s001.zip › Figure S4.tif]
